# Submerged leaves of live indoor foliage plants adsorb H1N1 influenza virus from suspension

**DOI:** 10.1080/15592324.2022.2163869

**Published:** 2023-01-12

**Authors:** Hak Jin Song, Sung Woo Yang, Jeong Wook Jo, Yong-Keun Choi, Im-Soon Lee, Byung Uk Lee, Sang Hyun Lee, Ho Hyun Kim, Kwang Jin Kim, Hyung Joo Kim

**Affiliations:** a Department of Biological Engineering, Konkuk University, Korea, South Korea; b Department of Biological Sciences, Konkuk University, Korea, South Korea; c Department of Mechanical and Aerospace Engineering, Konkuk University, Korea, South Korea; d Department of Nano-chemical, Biological and Environmental Engineering, Seokyeong University, Seoul, South Korea; e Urban Agriculture Research Division, National Institute of Horticultural and Herbal Science, Chungjoo, Korea

**Keywords:** *Epipremnum aureum*, influenza virus, plant-virus interaction, electrical grounding, rapid influenza diagnostic test kit, PFU

## Abstract

Control of hazardous indoor particles using plants has attracted interest due to the increasing worldwide air pollution and spread of pandemic-causing viruses. However, the interaction between human pathogenic viruses (HPVs) and live plants has not been examined largely due to issues in detecting tiny amounts of infectious viruses in a carrier (such as an aerosol) and the lack of suitable examination methods. In this study, as a novel evaluation method, the effect of submerged leaves of live plants on HPVs in water was examined, using the H1N1 influenza virus as a model. Selected plant foliage of a live plant was immersed in a small bag containing HPV water suspension. In an initial screening test, the activities of 20 different plant species on the virus suspension were evaluated using a rapid virus detection kit. Ten plant species had the capability to decrease virus concentrations in the water suspension within 72 h. Among the experimental plant species, *Epipremnum aureum* showed the highest virus decreasing characteristics when examined using both the kit and quantitative real time polymerase chain reaction. The capacity of immersed leaf of live *E. aureum* to decrease viral content was enhanced when the plant-containing pot was electrically grounded to the earth (approximately 70% decrease in virus concentration). The foliage sample analysis showed that virus adsorption to the plant foliage surface could be the major reason for the decrease in the suspension. These results suggest that the proposed method can be applied to select plants to further investigate plant–HPV interactions.

## Introduction

Recently, due to an increase in the time spent indoors and changes in lifestyle, the potential of control of indoor pollutants using living plants has attracted attention. Plants, an essential part of our living system, inevitably interact with various types of particles. Over the last decades, research has demonstrated that live plants interact with a wide range of small particles and chemicals, including airborne particulate matter,^1,2^ nanoparticles,^3^ volatile organic compounds (VOCs),^4^ airborne microbes,^5^ and other pollutants.^6^ The major known interactions between plants and these particles include absorption, adsorption, dilution, precipitation, and filtration.^4,7–9^ Using these interactions, which are known as phytoremediation, attempts have been made to control pollution in indoor environments.^10,11^ However, owing to the diversity of plant-related characteristics, such as differences in their rhizosphere, phyllosphere, metabolism, and foliage structure, the applicability of plants for phytoremediation for indoor pollution control has not yet been completely examined.^12^ Recent studies, therefore, have focused on the underlying phytoremediation mechanisms of the plant foliar system for hazardous particles.^4,8,11,13,14^ The surface and morphological characteristics of leaves of specific species are important parameters for the phytoremediation capacity of plants.^11,15^ Hence, understanding the phytoremediation mechanisms related to the removal of particles through plant shoot and root systems may provide a more versatile method for removing toxic materials, such as human pathogenic viruses (HPVs).

Phytoremediation of HPV using live plants has hardly been investigated due to the lack of practical specificity between these viruses and plants. Several studies have shown that foodborne HPVs suspended in the aqueous phase can attach to vegetable plant leaves.^16,17^ The attachment of HPVs on vegetable leaves, which might depend on epicuticular physicochemical properties, is closely related to the physiological activity of the plant.^16^ Experiments have been performed using isolated plant leaves (i.e., cut leaves), and, presumably, the water content, physiological activity, electrostatic forces, and surface properties of the experimental plant leaves differed from those of leaves connected to live shoot and root systems (i.e., a whole live plant in the soil system). Therefore, application of isolated plant leaves for experiments might provide limited or altered information regarding HPV–plant interactions. In addition, microbial contamination and decay of the isolated plant or plant leaves are inevitable and could pose major problems for the potential application for phytoremediation of HPV coupled with long-term indoor environment control. In this aspect, maintenance of plant activity during experiments can be assumed to provide more reliable and reproducible phytoremediation activity of plants for further applications. Reports have shown that during cultivation, experimental plant pots with electrical earth connections enhanced plant growth and activity compared to pots without earthing (i.e., conventional indoor plant pots that are electrically isolated from the earth – an infinite electricity sink).^18,19^ These reports suggested that electrical grounding of indoor plant pots provides geo-electrochemically friendly growth conditions for plants similar to those provided by plants that are connected (or grounded electrically) to the earth’s surface. Other reports have shown that plant growth and metabolism are closely related to the electromagnetic environmental conditions.^20,21^ Therefore, applying an electrical grounded connection to a live plant may induce stable physiological activity and could improve the phytoremediation activity of the plant.

Viruses are small infectious organisms that can be classified based on their genetic material. Their genetic, pathogenic, and spreading characteristics have been intensively studied. Recently, the rapid spread and infection of the severe acute respiratory syndrome coronavirus-2 (SARS-CoV-2) led to a global coronavirus disease-2019 (COVID-19) pandemic. Contact between plants, especially conventional indoor companion plants, and such HPVs in an enclosed space has a strong possibility. If the plant–virus contact is noticeable, plants could be further applied for indoor environment control. However, to the best of our knowledge, the contact between HPV particles and live indoor plants has not yet been studied, probably due to a dearth in suitable evaluation methods.

In this study, we describe a general method to estimate the interaction between HPV particles and live plants. Practical experiments were performed with HPV in the water suspension and submerged live plant foliage, and influenza H1N1 was used as a model HPV in this study. A reduction in the HPV levels in the suspension under various conditions, including with and without electrical grounding, was used as the basis of the experiments. The variations in HPV concentrations in the suspension were measured using a rapid influenza diagnostic test kit, along with quantitative real time polymerase chain reaction (qRT-PCR), and plaque assays. Additionally, we attempted to elucidate the behavior of the plant foliage on the HPV suspension by analyzing different parts of the experimental plant foliage.

## Materials and methods

### Plants and virus species used in the study

In this study, 20 different plants species (*Ardisia pusilla, Clusia rosea, Pelargonium inquinans, Dieffenbachia amoena, Syngonium podophyllum, Spathiphyllum wallisii, Peperomia obtusifolia, Euonymus japonicus, Zamioculcas zamiifolia, Radermachera sinica, Epipremnum aureum, Hedera helix, Philodendron tatei, Fatsia japonica, Zamia furfuracea, Monstera deliciosa, Strelitzia reginae, Hoya carnosa, Podocarpus nagi*, and *Anthurium andraeanum*) were examined for their effect of foliage on the H1N1 influenza virus particles suspended in water. The selection criteria for the experimental plants were easy indoor growing characteristics and foliage shape differences, based on previous work.^22^ All experimental plants were purchased from a local market (Yangjae Flower Market, Seoul, Korea). For a month prior to the experiment, the plants were cultivated in polyvinyl chloride (PVC) pots (200 × 180 mm diameter) containing 500 g of humus (Jeil, Myungga, Korea) under 130 μmol/m^2^/s illumination (light on/off ratio: 16/8 h) and at 22–25°C under 45–50% relative humidity (RH). The plants were watered with tap water (100 mL) every 3 d.

The H1N1 influenza virus (Influenza A/Human/Korea/KUMC-33/2006, 6.0 × 10^8^ plaque-forming units (PFU)/mL) used in this study was obtained from the Korea Bank for Pathogenic Viruses (Seoul, Korea). The test virus suspension was stored at −82°C. All virus-related experiments were performed at a Biosafety Level 2 laboratory at Konkuk University (Seoul, Korea).

### Plant–virus interactions

**Figure 1.**
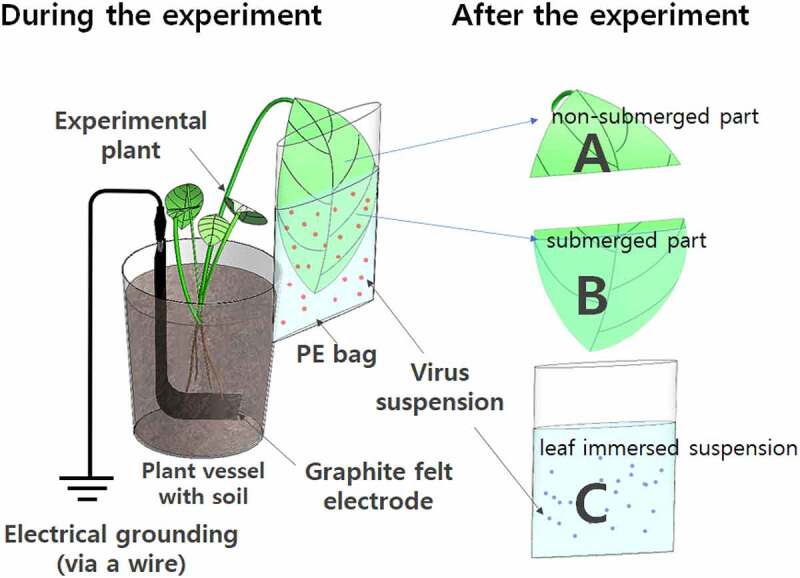
Schematic representation of the experimental system to evaluate the effect of plant on HPV suspension. The preliminary experiments were performed without electrical grounding, and the virus abundance in the leaf immersion suspensions (C) was analyzed using a rapid detection kit. In the case of *Epipremnum aureum*, samples from the virus suspension (C), submerged part of the leaf (B), and non-submerged part of the leaf (A) were analyzed using qRT-PCR (quantitative real-time polymerase chain reaction) with and without the electrical grounding conditions. In the illustrated example, graphite felt electrodes were used to electrically ground the plant pot.

Figure 1 shows a schematic diagram of the experimental system used in this study to evaluate the effects of submerged plant foliage on the suspended HPV. Briefly, the virus stock suspension was diluted with 1 mM sterilized phosphate buffered saline (PBS; final concentration: 6.0 × 10^5^ PFU/mL), and 3 mL of the suspension was transferred to a polyethylene bag (size: 6.5 × 10 cm). An undamaged, healthy leaf located approximately 60% below the top of the plant was generally selected for immersion in the virus suspension. The selected leaf was washed thoroughly with distilled water and then dried in a clean room for 12 h. After drying, a fixed area (both adaxial and abaxial surfaces: 16 ± 1.0 × 2 cm^2^) of leaf was immersed in the virus suspension in the bag. The top of the bag was then closed using paper tape to minimize evaporation during the experiment. A single leaf per experimental plant was selected for the experiment. The experiment was performed under 130 μmol/m^2^/s illumination (light on/off ratio: 16/8 h) at 25°C with 45–50% relative humidity (Figure 1). Approximately 1 h before the experiment, each plant was watered. After 72 h, 150 μL of the virus suspension (C in Figure 1) in the bag was harvested. The collected samples were then immediately assessed using the rapid influenza diagnostic test kit and qRT-PCR to evaluate the plant–virus interactions. For selected experimental species (i.e., *E. aureum*), two different experimental conditions were applied: plants in pots with electrical grounding, and plants in pots without electrical grounding. The experiment began when the *E. aureum* leaves were immersed in the virus suspension (final concentration: 6.0 × 10^6^ PFU/mL). After 72 h of immersion, qRT-PCR-based virus analyses were conducted on the samples of the virus suspension, submerged foliage, and non-submerged foliage (including the petiole; C, B, and A, respectively, in Figure 1). The virus suspension without leaf immersion was used as a control. All experiments were performed a minimum of three times, with different experimental plants of the same species. The averaged values are presented.

### Electrical grounding of plant pots

Plant pots containing the selected plant species (i.e., *E. aureum*) were connected to an electrical grounding point to examine the effect of the electrical grounding on the HPV suspension used in this study. The external earth point consisted of a copper bar (500 × 10 mm) inserted 500 mm into the wet ground outside the laboratory (earth ground resistance < 100 Ω; measured using a TK-2040 instrument, Taekwang, Korea). Among the electrode material, graphite felt was selected as an electrode material. Graphite felt, one of the most commonly used electrode material, was used in this study; it has a large effective surface area, which can increase the contact between microorganisms and graphite, thus inducing low internal resistance.^23^ Graphite felt electrodes (75 × 200 × 2 mm, GF series; GEE Graphite Ltd., UK) were positioned in plant pots to connect to the plant rhizospheres (Figure 1). The pots were initially filled with 300 g of humus (Jeil, Myungga, Korea) and fitted with the electrode at the surface of the filled humus. The plant roots were then placed against the surface of the electrode. The electrode and plant were then covered with additional humus (200 g). The electrical connections between the copper bar and plant pots consisted of copper wire (13 American wire gauge). An alligator clip and an earth clamp were used for the electrode-wire connection and the wire-ground point connection, respectively (Figure S1). For the control group (without grounding), the electrodes in the pots were not connected to the external earth point.^18^

### Determination of influenza virus concentrations using rapid influenza diagnostic test

For rapid determination of the presence and abundance of the influenza virus in the water sample, a rapid influenza diagnostic test kit (G10RHA20; Rapigen, Korea) was used. This kit provides a visual qualitative virus analysis result within 15 min, based on a viral antigen-antibody reaction.^24^ To verify the detection limit of the kit, serially diluted influenza virus suspensions were applied, and the optical signals from the kit were observed. The detection limit of the kit was observed in the range of 6 × 10^4^ to 6 × 10^5^ PFU/mL. Based on these observations, the virus concentration for the preliminary plant foliage application experiments was set at 6.0 × 10^5^ PFU/mL (Additional file 1: Figure S2).

### Determination of influenza virus concentration in liquid and solid samples using qRT-PCR

The viral abundance in aqueous suspension and foliage samples was quantitatively analyzed using qRT-PCR. For viral RNA extraction from the aqueous and solid samples, the HiGene™ Viral RNA/DNA Prep Kit (VN101-100; Biofact, Korea) was used. For aqueous phase samples, samples (150 μL; C in Figure 1) from the experimental system were used for RNA extraction. The fresh treated leaf samples (A and B in Figure 1) were first homogenized in 500 μL of 1 mM phosphate-buffered saline (PBS) using a sterilized mortar. All excessive leaf surface moisture and water droplets were removed using filter paper (No. 1; Whatman, USA) before homogenization. After homogenization, RNA was extracted using the kit. The extracted RNA sample (5 μL) was mixed with the amplification mixture (BioFACT™, qRT-PCR Master Mix for Probe; Biofact, Korea), which comprised 0.8 μM primer pairs (forward: 5’-AAG ACC AAT CCT GTC ACC TCT GA-3’ and reverse: 5’-CAA AGC GTC TAC GCT GCA GTC C-3’), 10 μL of the 2× PCR master mix, and 0.2 μM probe (FAM 5’-TTT GTG TTC ACG CTC ACC GT-3’ – TAMRA). Initially, reverse transcription was performed at 50°C for 30 min. After reverse transcription, the reaction was inactivated at 95°C for 15 min. The RNA-containing samples were then amplified for 40 cycles (1 cycle: 95°C for 20s and 61.2°C for 40s) with the amplification mixture in a PCR system (Gentier 96 real-time PCR system, China).^25^ All experiments were performed a minimum of three times.

### Determination of influenza virus concentrations in samples using plaque forming unit assay

To evaluate the virus activity in the samples, a plaque forming unit (PFU) assay was performed. The mammalian cell line (Madin–Darby Canine Kidney cell, MDCK) used in this study was obtained from the Korea Cell Line Bank (KCLB 10034, Seoul, Korea). The cell line was maintained with Eagle’s Minimum Essential Medium (EMEM, M4655, Sigma-Aldrich, USA) containing fetal bovine serum (FBS, S001-01, Welgene, Korea) and an antibiotic mixture (P4333, Sigma-Aldrich, USA) in a CO_2_ incubator (37°C, 5% CO_2_). For the plaque assay, the cells were cultivated in cell culture dishes (Model 20100, SPL Life Science, Korea) with 10 mL of the growth medium containing 1 mL of FBS and the 100 μL of the antibiotic mixture. The assay procedure was started when 90% cell adhesion was observed. The growth medium in the dish was removed, and the cells were washed twice with 6 mL of 0.1 M PBS. Then, the cells were released from the dish using 1 mL trypsin-EDTA (T4049, Sigma-Aldrich, USA) in a CO_2_ incubator for 15 min. To decrease the trypsin activity, 1 mL of the growth medium was added to the dish, and the number of cells in the released cell suspension was quantified using a hemocytometer (Model 0640110, Paul Marienfeld, Germany). For PFU analysis, 2 mL of the cell suspension (2.5 × 10^5^ cells/mL) was added to a 6-well cell culture plate (Model 30006, SPL Life Science, Korea). The suspension was then incubated in the CO_2_ incubator (37°C, 5% CO_2_) for 24 h. After cultivation, the attached MDCK cells were washed with 1 mL of 0.1 M PBS and then 0.4 mL of the sample from the plant system was added to the well. The plate was then incubated for 1 h to allow infection. The supernatant was removed from the wells, and the cells were washed with 1 mL of 0.1 M PBS. To identify the virus-infected MDCK cells in the well, 2 mL of EMEM with a 0.3% agarose mixture (9:1, v/v) containing 1 μg/mL trypsin (Model 4370285, Sigma-Aldrich, USA) was added to the wells. After 72 h of incubation, the cells were fixed with 1 mL of 0.1 M PBS containing 3.7% formaldehyde. After fixation, the cells were stained with crystal violet solution (MB80299, Kisan Bio, Korea) for 10 min. Any excessive staining solution was removed with tap water, and the number of plaques was quantified.^26^

## Results

### Preliminary screening of experimental plants using rapid influenza diagnostic test kit and qRT-PCR

The live foliage of 20 species of experimental plants was submerged and allowed to interact with the virus suspension (Figure 1) without electrical grounding. After 72 h, the virus suspensions (sample from “C” in Figure 1) were examined using the rapid influenza diagnostic test kit. Among the experimental species, 10 species produced negative signals (Table 1, Additional file 1: Figure S2). This result indicates that the presence of foliage of specific plant species affects the antigen-based virus concentration in the suspension. The remaining 10 plant species produced positive signals, indicating that the submerged leaves did not affect or reduce the virus concentrations to a specific level (6 × 10^4^ to 6 × 10^5^ PFU/mL). The negative and positive control groups (i.e., buffer solution only and virus suspension without leaf immersion, respectively) produced negative and positive signals, respectively (Additional file 1: Figure S2, top left and right, respectively). Therefore, the results suggest that the 10 plant species that produced negative signals had the ability to decrease the number of influenza virus particles to below 6.0 × 10^5^ plaque forming units (PFU)/mL in an aqueous solution. However, when the suspensions from the experimental plants were analyzed using qRT-PCR, viral RNA was detected in all plant foliage-associated suspension samples. The preliminary experiment (Additional file 1: Figure S3) revealed that the rapid influenza diagnostic test kit has a specific detection limit for the H1N1 virus. Therefore, the differences in the analytical results between the rapid influenza diagnostic test kit and qRT-PCR could be related to the differences in the detection sensitivity of these two methods. To maintain the accuracy of virus determination, the cycle threshold (Ct) values from qRT-PCR were used as the detection method and were converted to PFUs using a pre-established virus concentration calibration curve (Additional file 1: Figure S4).

**Table 1.** Interaction between an influenza virus in aqueous suspension and submerged plant leaves of various species.

Plant species	Detection of viral antigen (+ positive, – negative)
*Ardisia pusilla*	-
*Clusia rosea*	+
*Pelargonium inquinans*	-
*Dieffenbachia amoena*	-
*Syngonium podophyllum*	-
*Spathiphyllum wallisii*	-
*Peperomia obtusifolia*	+
*Euonymus japonicus*	-
*Zamioculcas zamiifolia*	+
*Radermachera sinica*	+
*Epipremnum aureum*	-
*Hedera helix*	-
*Philodendron tatei*	+
*Fatsia japonica*	-
*Zamia furfuracea*	+
*Monstera deliciosa*	+
*Strelitzia reginae*	+
*Hoya carnosa*	-
*Podocarpus nagi*	+
*Anthurium andraeanum*	+
Virus (Positive Control)	+
1 mM PBS (Negative Control)	-

PFU of the virus suspension: 6 × 10^5^. The symbols indicate the results of a rapid influenza diagnostic test (+ positive, – negative). PFU: Plaque forming unit; PBS: Phosphate-buffered saline.

**Figure 2.**
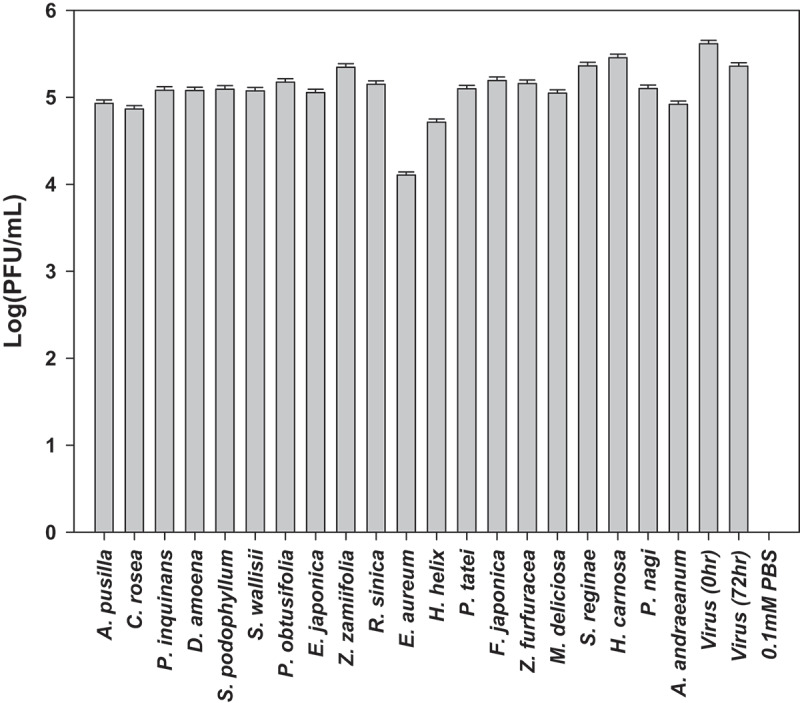
Variations in virus concentration in suspensions after immersion of various species of plant foliage. The initial virus concentration was 6.0 × 10^5^ plaque-forming units (PFU). The immersions were maintained for 72 h, and the residual suspensions (C in Figure 1) obtained from the plants were analyzed using qRT-PCR. Virus (0 h) and virus (72 h) indicate the viral suspensions at the initial stage of the experiment and after 72 h of immersion, respectively. Negative control: 1 mM PBS. The PFUs were converted using a pre-established Ct-PFU calibration curve (Fig. S4). Error bars represent standard deviations of at least three experimental runs.

When the qRT-PCR results from the experimental plants were evaluated, the lowest H1N1 virus concentration was observed in the *E. aureum* leaf suspension (Figure 2, Additional file 1: Figure S5), indicating that live *E. aureum* leaf has the ability to decrease the qRT-PCR-based number of H1N1 viruses in the suspension. In addition to *E. aureum*, most of the other species that produced negative signals in the rapid influenza diagnostic test (*Ardisia pusilla, Pelargonium inquinans, Dieffenbachia amoena, Syngonium podophyllum, Spathiphyllum wallisii, Euonymus japonicus, Hedera helix, Fatsia japonica*, and *Hoya carnosa*) led to a decrease in the virus concentration (Additional file: Figure S5). However, of the above species, *F. japonica* and *H. carnosa* produced no significant differences in the qRT-PCR results compared to control (virus incubation for 72 h without plant leaf immersion). Ten species (*Clusia rosea, Peperomia obtusifolia, Zamioculcas zamiifolia, Radermachera sinica, Philodendron tatei, Zamia furfuracea, Monstera deliciosa, Strelitzia reginae, Podocarpus nagi*, and *Anthurium andraeanum*) showed positive signals in the rapid influenza diagnostic test. These results indicate no consistent relationship between antigen- (i.e., the rapid detection kit) and RNA-based virus detection (i.e., qRT-PCR) for analyzing the plant–virus interactions. As *E. aureum* showed clear and consistent signals in both the rapid influenza diagnostic test and qRT-PCR, we selected *E. aureum* for further experiments related to elucidating the effect of live plants on H1N1 virus suspension.

## Effect of live *E.* *aureum* foliage on H1N1 virus water suspension

**Figure 3.**
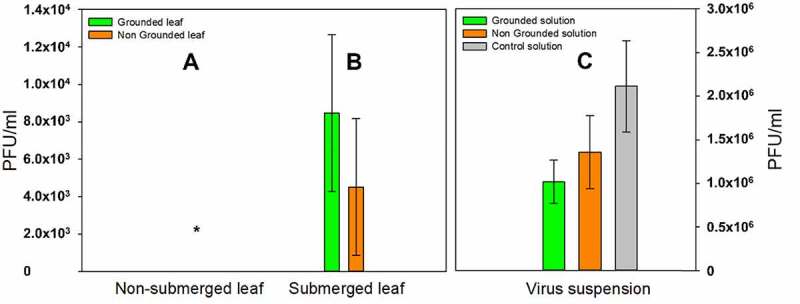
Effect of *E. aureum* leaf immersion on aqueous phase virus suspension. The initial virus concentration for the experiment was 6 × 10^6^ PFU/mL. Leaf immersions were maintained for 72 h, and the virus concentrations in the leaf samples (A and B in Figure 1) and suspension (C in Figure 1) were analyzed using qRT-PCR. A, B, and C, as in Figure 1. The asterisk (_*_) indicates that no virus was detected in any of the samples in the non-immersed part of the leaves of the ground-connected, non-ground-connected, and dried leaf samples. The PFU values used in this figure were converted from a pre-established calibration curve (Additional file 1: Fig. S4). Grounded solution: virus suspension with immersion of a grounded plant leaf; Non Grounded solution: virus suspension with immersion of a non-grounded plant leaf; Control solution: virus suspension without the immersion of a plant leaf; Grounded leaf: grounded plant leaf; Non Grounded leaf: non-grounded plant leaf. Error bars represent standard deviations of at least three experimental runs.

To evaluate the effect of the live plant against the suspended H1N1 virus, the leaf of *E. aureum* was submerged in the virus suspension for 72 h. The variations in the number of viral particles in the submerged leaf-associated suspensions (C in Figure 3), under different experimental conditions, were analyzed. The highest PFU was observed in the control experiment (i.e., H1N1 suspension without plant leaf immersion; C in Figure 3). However, approximately 60% of the H1N1 viruses were lost during the experimental period in which plant leaves were not immersed. The stability and infectivity of viruses are reportedly affected by sunlight, presence of bacteria, and temperature conditions.^27^ The experimental environments (illumination and temperature) might have naturally induced this virus reduction in the control experiment. When the H1N1 suspensions with *E. aureum* leaves were monitored, both the electrically grounded and non-grounded plants induced comparably higher decrease in virus concentrations as quantified by qRT-PCR (70% decrease in the grounded plant and 50% decrease in the non-grounded plant compared to the control). This result suggested that the plant leaf has an ability to reduce the influenza virus concentration in water and indicates that grounding the plant induced an improvement in the virus reducing action.

A and B in Figure 3 shows the virus concentration per mL of the homogenized experimental leaves. The highest PFU was observed on the electrically grounded leaf. Although H1N1 viruses were also present on the non-grounded leaf, their concentrations were relatively low. These results could partially explain why the lowest amount of H1N1 was found in the suspension with the electrically grounded plant foliage. During leaf immersion, an electrical connection to the plant pot may promote virus adsorption on the leaf surface and decrease the number of viral particles in the suspension. When the non-submerged part of the leaf (sample “A” in Figure 3) was analyzed using qRT-PCR, no viruses were observed, irrespective of the experimental conditions (i.e., grounded and non-grounded). This finding suggests that viral particles in the suspension were adsorbed to the leaf surface and barely entered the stomatal systems of the plants.^28,29^ In addition, notably, the number of viral particles in the immersed leaf suspensions and control suspension (i.e., liquid samples) was not comparable to that of the solid leaf samples. In the grounded plants, for example, approximately 1.5 × 10^6^ PFU/mL of the virus was lost during immersion. However, only 6.0 × 10^3^ PFU/mL were found on the solid leaf sample. A possible reason for this difference is the degradation of viral RNA during the qRT-PCR sample treatment (the drying and homogenization processes). Several potential antiviral chemicals^30–32^ may have been released from plant tissues, especially during homogenization, which have induced serious errors in the particle determination of the influenza virus in the foliage samples. These phenomena may explain the nonexistence of PFU in the non-submerged parts of the foliage (A in Figure 3).

When a plaque assay was performed on samples from all experimental groups, countable number of plaques were only observed in the control samples (i.e., virus suspension only) at the onset of the experiment (Additional file 1: Figure S4). Samples of the leaf-immersed suspensions and homogenized plant foliage showed no plaque formation on the mammalian cell line, potentially due to a loss of infectivity during the interaction period and the subsequent sample treatment processes. Therefore, the plaque assay was only applied to partially verify the calibration curve (Additional file 1: Figure S3).

## Discussion

The application of indoor plants for the control of air contaminants, such as VOCs and particulate matter, has been the focus of research due to the recent public health-related concerns during the COVID-19 pandemic. Several reports have shown that the spread of aerosols is a major transmission route for HPV.^33–35^Therefore, various attempts have been made to elucidate the interaction between airborne HPV and plants. However, owing to difficulties in virus detection and analysis (e.g., detection limit, detection time, and sample amount), research has been restricted to foodborne HPV.^17,36^ The diffusion of minute virus-contaminated airborne water droplets or aerosols in an enclosed space is a critical reason for the spread of pathogenic virus-based diseases, such as influenza and COVID-19.^37^ Currently, the application of molecular biology techniques, such as qRT-PCR, for virus detection has led to important breakthroughs; however, applying this method for a small amount of virus samples adsorbed on live plant leaves or for a volume of a single aerosol is challenging. Although the plant leaf immersion method used in the present study may not completely elucidate the interaction between small H1N1-containing airborne particles (e.g., aerosols) and plant foliage surfaces, the method described in this study was aimed at establishing a basic research technique to investigate the effects of plant behavior on HPV and this method should be viewed as a “broad brush” indicator for the control capacity of plants against HPVs with a useful, simple examination procedure.

With electrical grounding, a relatively higher PFU was expected to be transferred from the suspension (C in Figure 1) to the submerged leaf (B in Figure 1). Previous research^18^ has shown that electrical grounding of plants induces higher germination and growth rates, and the grounding of plant pots provides a suitable “electrochemical environment” resembling cultivation on natural soil. Without electrical grounding of the plant (for instance, an electrically isolated indoor plant), the accumulation of charged molecules on the plant surface may negatively affect the growth of the plant, due to the hazardous range of electricity.^38–40^ In practice, electrical grounding allows any excess electrical charge to drain away. The earth, which represents an infinite electrical ground, can absorb an unlimited amount of electrical charge. Therefore, during plant growth, the presence and absence of electrical grounding may alter unknown electrochemical factors, thereby inducing alternations in plant metabolism and the surface charge of foliage systems. In the present study, a larger decrease in H1N1 virus titer was observed with the electrically grounded plant foliage. This result might be related to plant activation, changes in the surface charge of plant leaves, and/or the drain of accumulated electric charges, due to the grounded connection.

Analysis of the submerged part of the plant foliage (B in Figure 1) provided imprecise information on the adsorption or absorption (or both) of the virus by the plant foliage system. Several studies have used inductively coupled plasma-mass spectrometry (ICP-MS) to monitor the behaviors of highly stable metal-based particles in the plant system. Such studies have shown that absorption of nano-sized particles (ca. 20–60 nm in diameter) by plant foliage is possible.^28,29^ Therefore, during the leaf immersion stage of this experiment, some virus particles may have been absorbed by the stomatal system of the plant. In addition, during the absorption process in the current study, viral material may have been destroyed or inactivated by plant molecules, such as enzymes and cellular components.^41,42^ The current study of the possible absorption of active virus material by a plant system is further complicated by the extremely small size and complex structure of active HPVs, which make their experimental investigation in the plant system difficult. In particular, during foliage homogenization for sample preparation, the viral RNA for the qRT-PCR could have been mixed with plant molecules, possibly inducing serious errors in the plaque assay counts. These phenomena may explain the low PFU counts in the submerged part of the foliage (B in Figure 1) and the nonexistence of PFU in the non-submerged parts of the foliage (A in Figure 1). In the present study, a single concentration of virus suspension was used during the 72 h of plant immersion; however, different virus concentrations and interaction times may lead to differences in the mechanisms and rates of adsorption or removal (or both) of viruses by the submerged plant foliage. In addition, different plant species may have more efficient virus removal activity in suspension, as exudates may be secreted from active plant foliage after an extended period of immersion in a liquid.^43^ However, the secretion of exudates or chemicals from the live plant foliage during immersion was not considered in the present study. Therefore, further analysis of the interaction between immersed leaves and virus suspensions may provide interesting information related to the behavior of plants and influence of plant leaf exudates on the presence of HPV on the surface of plant leaf.

The current study evaluated the effects of the submerged live *E. aureum* foliage on suspended H1N1 virus particles. Although the *E. aureum* foliage showed the highest virus decreasing effect, other plant species also showed virus decreasing behavior in suspension. The reason for the comparatively strong HPV-concentration-decreasing effect produced by water-submerged *E. aureum* foliage is not clear. However, this result is tentatively attributed to the high hydrophilicity that is related to the low wax quantity at the leaf surface.^44^ Notably, the plant species used in the present study exhibit different physiological characteristics and may show variations in leaf surface structures, morphologies, and stomata size and density.^45^ In addition, the immersion area of the foliage of specific plant species may affect the plant–virus interaction, owing to changes in the physiological activity of the plant, including stomatal pore opening size.^46^ Therefore, under optimal conditions, more effective virus removal by the foliage of these plant species could be observed.

The spread of aerosols is a major transmission route for HPV.^33–35^ Therefore, development of physical and chemical airborne HPV removal systems, including ionization, UV radiation, and biological filters, have been recognized as essential for virus control.^37^ However, no airborne HPV control method involving plant activity has been reported yet. Therefore, the method described in the present study allows the assessment of the effects of plants on various HPVs by a comparison of plant and virus species through easy procedures for the application of plant and HPV related research and environmental controls.

In summary, the current study evaluated the effect of submerged live plants on an HPV water suspension as a novel method. Although the *E. aureum* foliage showed the highest HPV decreasing effect, presumably by adsorption, other plant species also showed the same effect in suspension. The electrical connection between the plant cultivating pot and earth point enhanced the HPV-adsorption activity of the plant. The present study focused only on the effect of submerged plant leaves on H1N1 viruses present at high concentrations in water but did not examine the effect on virus-containing aerosols. Therefore, our interpretation of plant–HPV interactions is limited. Further studies with improved experimental settings are required to better understand the mechanisms underlying the plant–virus interactions in the presence of electrical grounding and the migration of various virus species within plant systems using controlled environments. Such future studies should focus on the effect of electrical grounding on the opening and closing of stomata, measuring the electric potential of leaf surfaces, the adsorption and absorption of HPV by plants, the behavior of adsorbed viruses, and the effects of different plants on HPV.

## Supplementary Material

Supplemental Material
