# Supplementary material for: Submerged leaves of live indoor foliage plants adsorb H1N1 influenza virus from suspension
Source: Plant Signal Behav. 2023 Jan 12;18(1):2163869. doi: 10.1080/15592324.2022.2163869 (PMC9851199; doi:10.1080/15592324.2022.2163869)
Supplement: Supplemental Material [file KPSB_A_2163869_SM6554.docx]

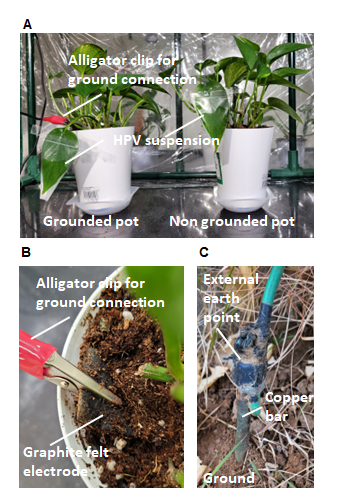
**Supplementary information**

Figure S1. Photograph of the plant–HPV interaction experimental system (A), the electrode-wire connection (B), and the external ground point (C) used in this study.


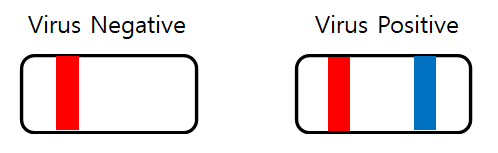

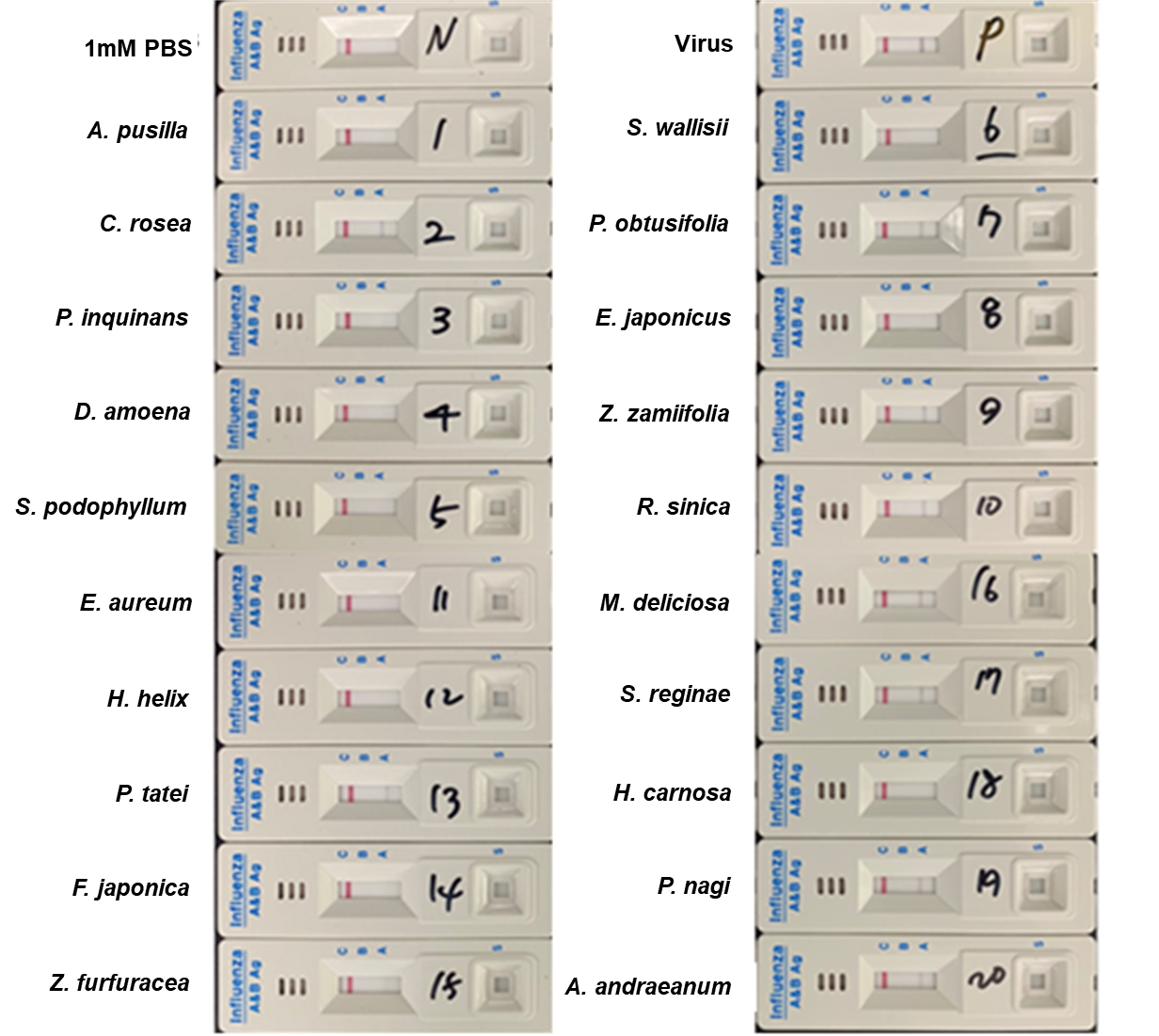


Figure S2: The results of the preliminary analysis of plant–virus interactions using the rapid influenza diagnostic test kit.


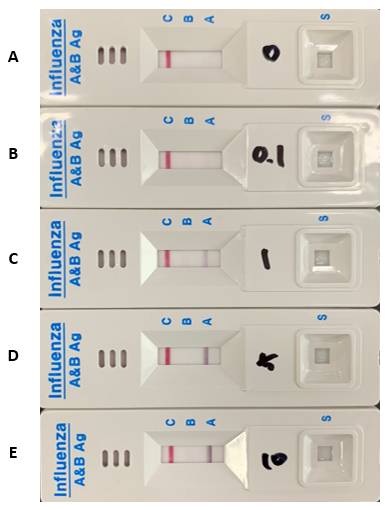


0 PFU/mL

6 x 10^4^ PFU/mL

6 x 10^5^ PFU/mL

3 x 10^6^ PFU/mL

6 x 10^6^ PFU/mL

**Virus concentrations**

**Rapid kit results**


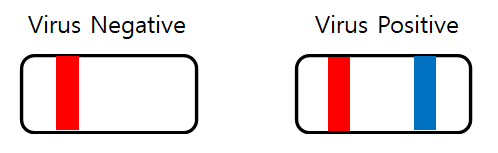


Figure S3. Estimation of the detection limit of the rapid influenza diagnostic test kit for influenza H1N1 virus suspensions.


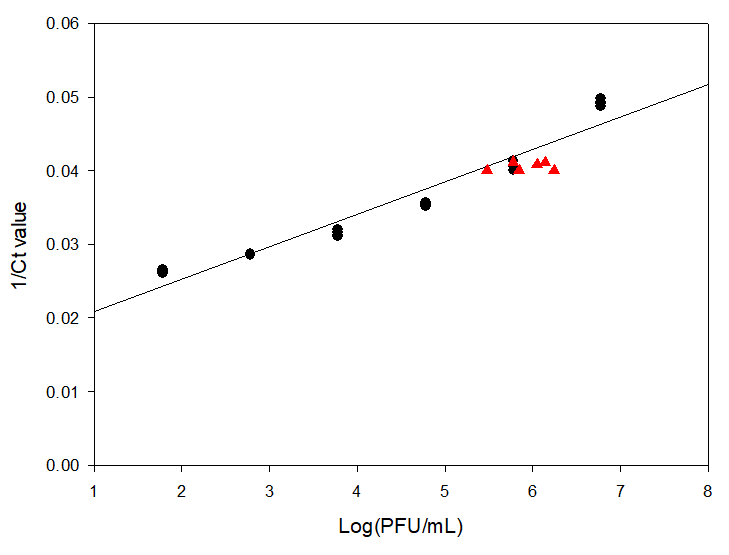


Figure S4. Calibration curve for PFU and Ct value conversion. The Ct values were obtained from the diluted virus suspension using qRT-PCR (●). A plaque assay was also performed, and the corresponding qRT-PCR results are plotted on the graph (▲). Plaque assay results (6.0 × 106 PFU/mL) are shown in the figure. The estimated correlation coefficient (r2) of the graph is 0.94.


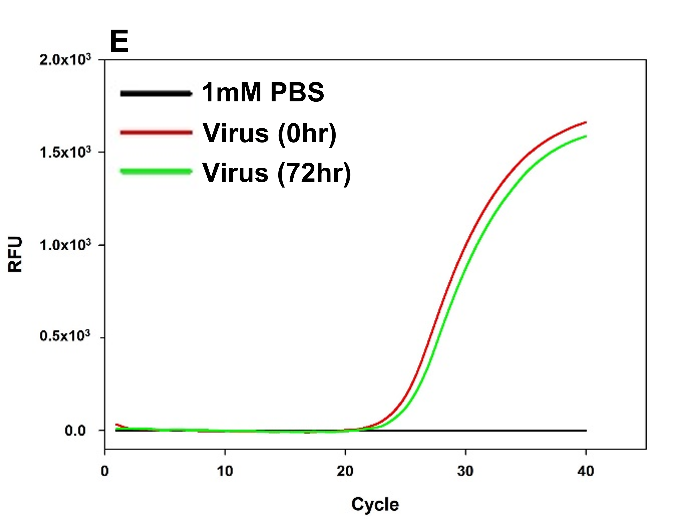

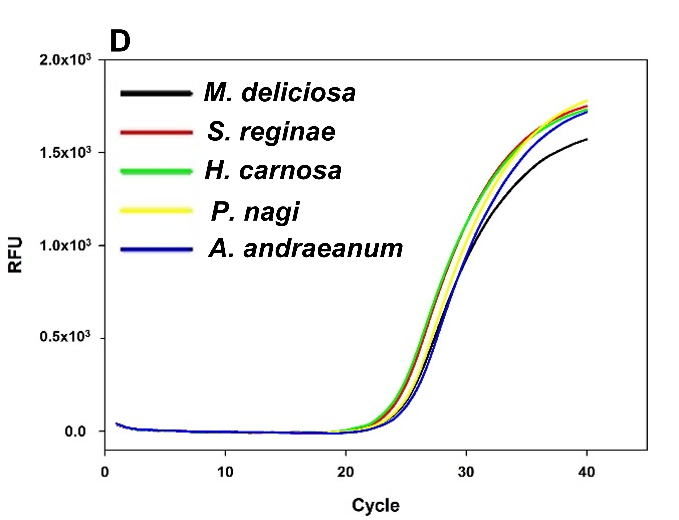

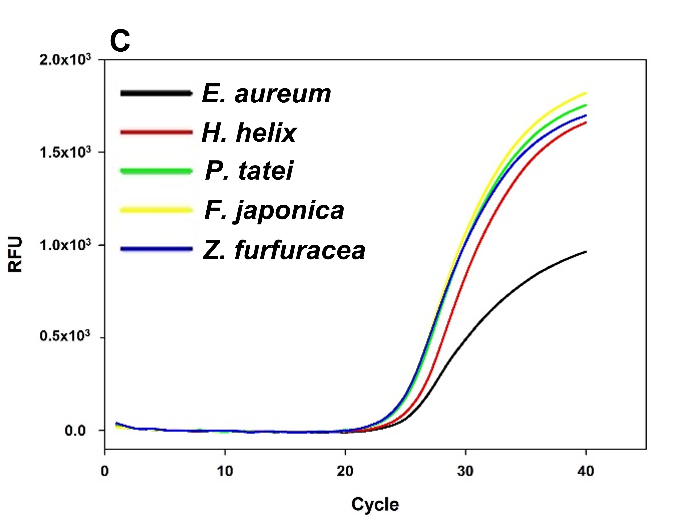

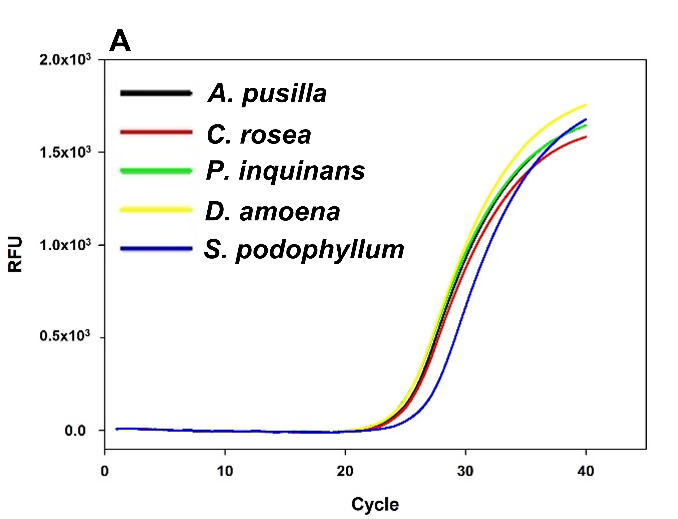

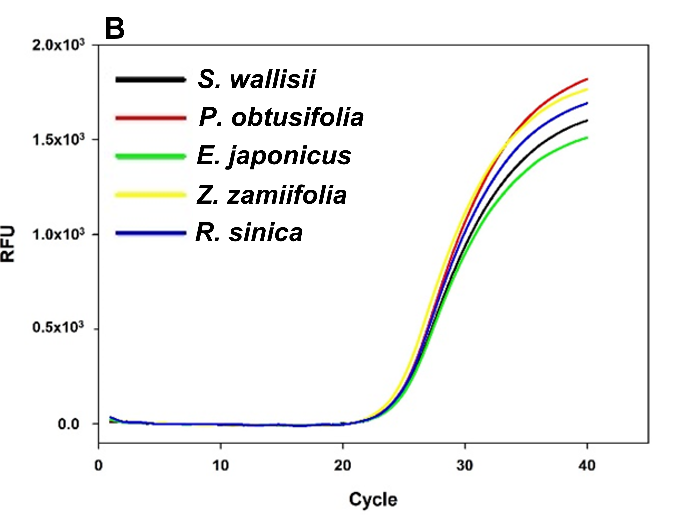


Figure S5. qRT-PCR results (RFU, Relative Fluorescence Units) from the various immersed-plant virus suspensions after 72 h of interaction. A (*A. pusilla*, *C. rosea*, *P. inquinans*, *D. amoena*, *S. podophyllum*), B (*S. wallisii*, *P. obtusifolia*, *E. japonicus*, *Z. zamiifolia*, *R. sinica*), C (*E. aureum*, *H. helix*, *P. tatei*, *F. japonica*, *Z. furfuracea*), D (*M. deliciosa*, *S. reginae*, *H. carnosa*, *P. nagi*, *A. andraeanum*), E (1 mM PBS, Virus (0hr), Virus (72hr)).


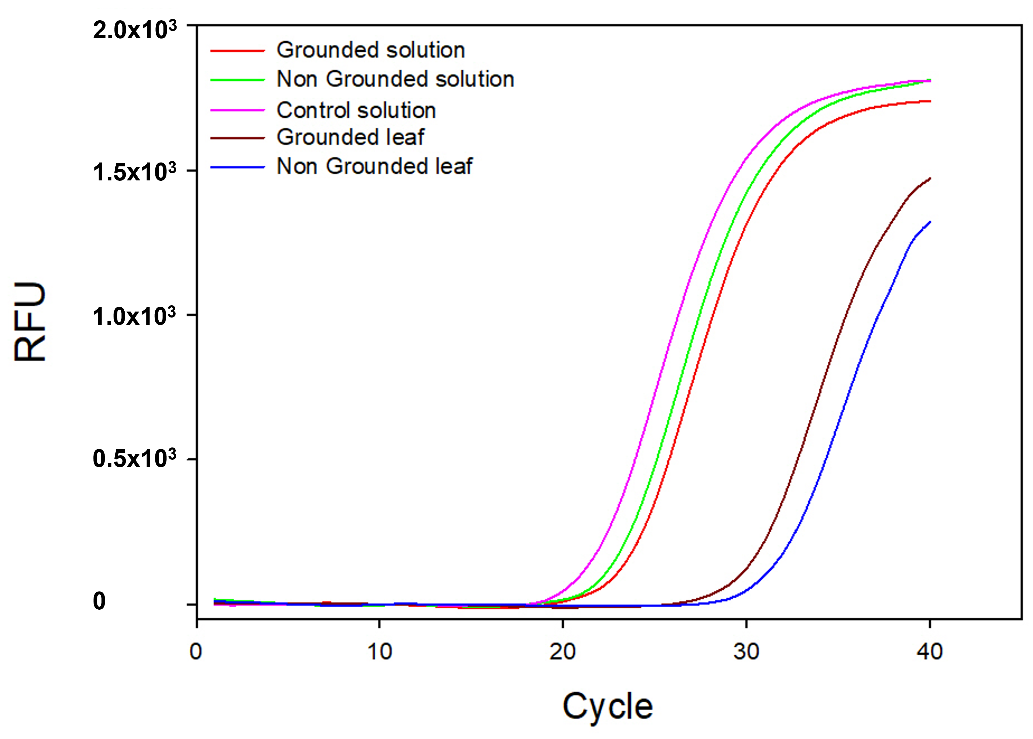


Figure S6. qRT-PCR results (Ct) from the experimental plant (*E. aureum*) and plant-immersed virus suspensions after 72 h of interaction.
